# Ticks infesting humans and associated pathogens: a cross-sectional study in a 3-year period (2017–2019) in northwest Italy

**DOI:** 10.1186/s13071-021-04603-x

**Published:** 2021-03-05

**Authors:** Tania Audino, Alessandra Pautasso, Veronica Bellavia, Valerio Carta, Alessio Ferrari, Federica Verna, Carla Grattarola, Barbara Iulini, Maria Domenica Pintore, Mauro Bardelli, Germano Cassina, Laura Tomassone, Simone Peletto, Valeria Blanda, Alessandra Torina, Maria Caramelli, Cristina Casalone, Rosanna Desiato

**Affiliations:** 1 Istituto Zooprofilattico Sperimentale del Piemonte, Liguria e Valle D’Aosta, Turin, Italy; 2 Universita’ Degli Studi di Torino, Turin, Italy; 3 Azienda Sanitaria Locale del Verbano Cusio Ossola (ASL VCO), Omegna, Italy; 4 Azienda Sanitaria Locale di Asti (ASL AT), Asti, Italy; 5 Azienda Sanitaria Locale 1 Imperiese (ASL1 Imperiese), Sanremo, Italy; 6 Istituto Zooprofilattico Sperimentale della Sicilia “A. Mirri”, Palermo, Italy

**Keywords:** Tick-borne diseases, PCR, *Rickettsia* spp., *Borrelia* spp., *Anaplasma phagocytophilum*

## Abstract

**Background:**

Tick-borne diseases are common throughout Europe. Ticks transmit pathogens to the host while feeding and together with mosquitoes, they are major vectors of infectious agents worldwide. In recent years, there has been a marked increase in the incidence of tick-bite events and tick-borne disease in northwest Italy, but information on the prevalence of tick-borne pathogens in ticks removed from humans remains scarce. To fill this gap, we report here the prevalence of tick bites and tick-borne pathogens documented for humans in Piedmont, northwest Italy, in the 3-year period 2017–2019.

**Methods:**

Ticks attached to humans during 2017–2019 were collected from residents of urban and rural area by physicians and veterinarians working with local veterinary agencies. All ticks (*n* = 1290) were morphologically identified to the species level. A subset of ticks removed from children (age 0–18 years) and the elderly (> 70 years), both age groups considered to be at-risk populations, was screened by biomolecular analysis to detect pathogens (e.g. *Rickettsia* spp., *Borrelia* spp., *Anaplasma* spp.). Pathogen identity was confirmed by Sanger sequencing.

**Results:**

Ticks were taxonomically assigned to ten species of six genera (*Amblyomma*,* Dermacentor*,* Haemaphysalis*,* Hyalomma*,* Ixodes* and *Rhipicephalus*). Most belonged to the genus *Ixodes*: 1009 ticks (78.22%) were classified as *Ixodes ricinus*. A subset of 500 ticks collected from the two at-risk populations were subjected to PCR assay to determine the presence of *Rickettsia* spp., *Borrelia* spp., and *Anaplasma* spp. The overall prevalence of infection was 22.8% (*n* = 114; 95% confidence interval [CI]: 19.19–26.73%), meaning that at least one pathogen was detected: *Rickettsia* spp. (prevalence 15%, *n* = 76; 95% CI 12.17–18.65%); *Borrelia* spp. (prevalence 6.4%, *n* = 32; 95% CI 4.42–8.92%); and *Anaplasma* spp. (prevalence 1.2%, *n* = 6; 95% CI 0.44–2.6%).

**Conclusions:**

Our data underline the importance of surveillance in the epidemiology of tick-borne diseases and the implementation of strategies to control tick infestation and associated pathogens.
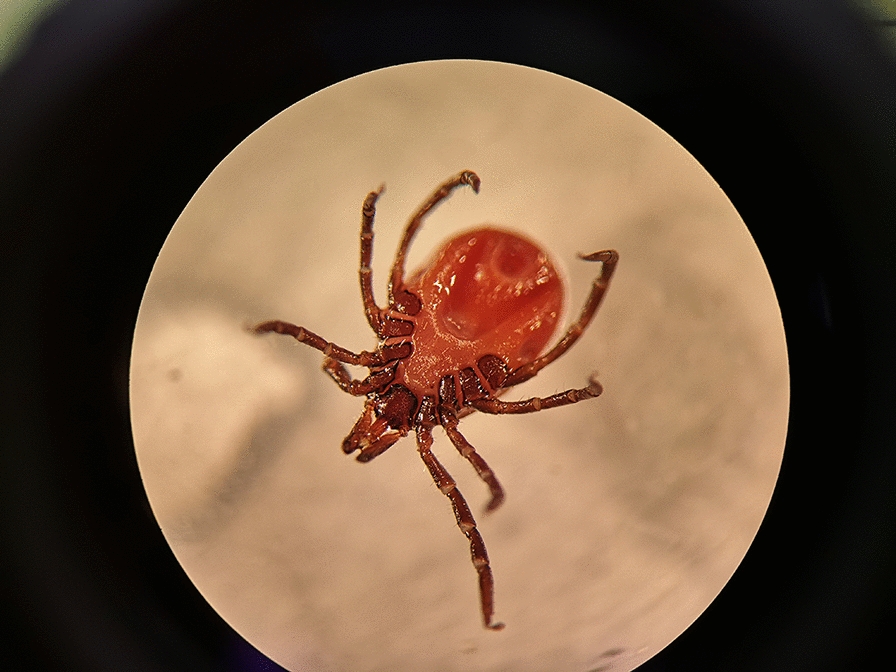

## Background

Ticks are major vectors of zoonotic pathogens in temperate regions [16]. They have a worldwide distribution owing to their ability to adapt to diverse environments, climate zones and host species [9, 17]. In addition, they transmit a variety of pathogens of medical and veterinary importance (e.g. viruses, bacteria, protozoans, helminths) that are responsible for a diverse range of infections, commonly referred to as tick-borne diseases (TBDs) [38]. Many TBDs are zoonoses, such as rickettsiosis, Lyme borreliosis, anaplasmosis [1] and tick-borne encephalitis (TBE), which may remain asymptomatic or manifest with potentially life-threatening involvement of the central nervous, the integumentary or the vascular system. Children, the elderly and the immunosuppressed are at higher risk of developing severe illness.

Rickettsiosis is a bacterial disease caused by obligate, intracellular α-proteobacteria of the genus *Rickettsia*. These Gram-negative, pleomorphic bacteria are prevalent in Sicily, Sardinia, Latium, and Calabria regions of Italy. Until 2002, *Rickettsia conorii conorii*, identified as the causal agent of Mediterranean spotted fever (MSF), was the only pathogenic *Rickettsia* species in Italy. A number of new *Rickettsia* species have since been identified by molecular analysis which have been described and recognized as causative agents of human disease in Europe. Twenty-six *Rickettsia* species with validated and published names are currently reported worldwide [29].

Spirochetes of the complex *Borrelia burgdorferi* (*s.l.*) are the etiological agent of Lyme borreliosis. Lyme disease typically presents as an erythema migrans rash and non-specific symptoms (e.g. fatigue, fever, headache, muscle and joint pain), but if left untreated it can progress to multisystemic disease. Though rarely fatal, deaths linked to Lyme carditis have been reported [35] (Kugeler et al. 2011). The disease is prevalent in Friuli-Venezia Giulia, Veneto, Trentino-Alto Adige (all regions in northeast Italy), Liguria (northwest Italy) and Emilia-Romagna (central Italy), whereas *B. burgdorferi* is reported only sporadically in both humans and ticks in the south-central regions of Italy and the islands (EpiCentro; https://www.epicentro.iss.it/zecche/borreliosi).

*Anaplasma phagocytophilum* is responsible for granulocytic anaplasmosis. It may be asymptomatic or cause non-specific symptoms (e.g. fever, headache, muscle ache). The fatality rate is < 1% [2, 11].

The worldwide incidence of TBDs has increased [9] in parallel with the survival and spread of vectors. Local climatic factors (macro- and microclimate) in addition to environmental factors can facilitate the appearance or reappearance of vector-borne diseases in a given area [17]. The distribution and prevalence of TBDs are closely related to climate factors, primarily high and low temperature extremes and precipitation patterns. Climate change can modify weather patterns, leading to an increase in extreme events and disease outbreaks by altering biological variables, including vector population size and density, vector survival rates, relative abundance of reservoir hosts and pathogen reproduction rates [14]. Collectively, these changes can increase the risk of pathogens being transmitted to humans.

The Mediterranean regions have a remarkable geographical and wildlife diversity, with high environmental variability resulting from the influence of altitude and distance from the sea. The variability of environmental characteristics favors the formation of tick populations. Italy has more tick species (about 40 species; [21]) than any other European country, including Portugal [32] and the UK [34, 37]. By virtue of its geographical extension from the Alps in the north to the Mediterranean in the south, Italy has a wide range of diverse habitats and given its geographical location in the north/south migration path of wild birds from Africa to Europea, it provides a port of entry for the arrival of new pathogens.

The incidence of human TBDs in Italy is likely underestimated due to poor surveillance and the limited number of available studies. Since 2011, the Istituto Zooprofilattico Sperimentale of Piedmont, Liguria and Valle d’Aosta (IZS PLVA), a public health agency, has conducted TBD surveillance in Italy. Ticks are identified morphologically to the species level and subjected to biomolecular analysis (PCR) for the detection of *Rickettsia* spp.,* Borrelia s*pp. and *Anaplasma* spp. Here, we report data from surveillance carried out during the 3-year period 2017–2019 in northwest Italy. The data will be used for further risk assessment.

## Methods

### Tick collection and identification

A total of 1290 ticks were collected, of which a subsample from two at-risk populations (children aged < 18 years and adults aged > 70 years) were tested for the presence of pathogens of the genera *Rickettsia*,* Borrelia* and* Anaplasma*. Most samples (*n* = 1254) came from the geographical areas falling under the administration of IZS PLVA or from people who had traveled abroad (*n* = 12) or were from neighboring regional areas (*n* = 14); some were of unknown origin (*n* = 10). All specimens were kept in 70% undenatured alcohol or frozen at − 20 °C and sent to the IZS PLVA laboratories for analysis. Species identification was performed using appropriate taxonomic keys [12] for each developmental stage (larvae, nymphs, adult female or male).

### Molecular analysis

#### DNA extraction

Tick DNA was extracted from adults, nymphs or larvae. Each microtube containing a tick was filled with either 350 μl (larvae and nymphs) or 600 μl (adults) of phosphate buffered saline solution (pH 7.2). The ticks were then homogenized using a Savant FastPrep FP120 cell disrupter (Thermo Fisher Scientific, Waltham, MA, USA) for 45 s at maximum speed (6.5 m/s). After homogenization, the microtubes were centrifuged (MIRKO 22R; Andreas Hettich GmbH & Co. KG, Tuttlingen, Germany) for 10 min at 14,000 rpm, and 150 μl of the supernatant was used for total DNA extraction with a QIAamp DNA Mini kit with an automated QIAcube protocol and following the manufacturer’s instructions (Qiagen, Hilden, Germany).

#### PCR assays

The PCR assays were performed in a total volume of 25 µl according to previously published protocols [7, 22, 36] for the detection of *Rickettsia* spp., *Borrelia burgdorferi* (*s.l.*) complex and *Anaplasma* spp. PCR amplification was followed by gel electrophoresis on 2% agarose and visualization with GelRed staining under UV light. The target genes, primer sequences and expected amplicon sizes are reported in Table 1.

**Table 1:** Molecular detection of tick-borne pathogens: target genes, primer nucleotide sequences, amplicon size

Species	Target gene	Nucleotide sequence (5′–3′)	Amplicon size (bp)	Reference
*Rickettsia* spp.	*ompB*	GTAACCCGGAAGTAATCGTTTCGTAA (forward) GCTTTATAACCAGCTAAACCACC (reverse)	511	[7]
*Borrelia burgdorferi* (*s.l.*)	*Flagellin*	AGAGCAACTTACAGACGAAATTAAT(forward) CAAGTCTATTTTGGAAAGCACCTAA (reverse)	482	[36]
*Anaplasma phagocytophilum*	*msp2*	CCAGCGTTTAGCAAGATAAGAG (forward) GMCCAGTAACAACATCATAAGC (reverse)	334	[22]

### DNA sequencing

The PCR products were purified using a QIAquick Gel Extraction kit (Qiagen). The cycle sequencing reaction was following the protocol of the BrilliantDye Terminator v.3.1 kit (NimaGen, Nijmegen, The Netherlands; 1 µl BrilliantDye v 3.1, 3.5 µl 5× sequencing buffer, 1 µl template, 1 µl primer 5pMol, 13.5 µl water) using the PCR primers for sequencing. The cycle sequencing reactions were purified with an AutoSeq G-50 Dye Terminator Removal kit (GE Healthcare, Chicago, IL, USA). Sanger sequencing was carried out by capillary electrophoresis using a 3130XL Genetic Analyzer (Applied Biosystems, Foster City, CA, USA). Obtained sequences were compared using the basic local alignment search tool (BLAST) provided by the National Center for Biotechnology (http://blast.ncbi.nlm.nih.gov/), with sequence records available in GenBank for confirmation of pathogen identification and species assignment.

### Statistical analysis

Statistical analysis was performed using Stata Statistical Software, Release 15.1 (StataCorp LP, College Station, TX, USA), including creation of tables and calculation of prevalence. QGIS 2.18, a geographic information system application, was used to visualize and create the map showing the tick distribution in the three regions of the country. A non-parametric test was employed to investigate the association between tick number and bite frequency. As the distribution of number of ticks was skewed, the median test (StataCorp LP) was applied. Covariates showing a significant association with the number of ticks were categorized and entered into a subsequent linear regression model after log-transformation of the dependent variable (number of ticks).

## Results

A total of 1290 ticks were collected from humans in the period 2017–2019 and morphologically identified, of which 239 (18.5%), 624 (48.4%) and 427 (33.1%) were collected in 2017, 2018 and 2019, respectively. A peak in the number of ticks was observed in 2018 due to a persistent warm spell in the spring (warm and wet weather conditions; https://www.arpa.piemonte.it/rischinaturali/tematismi/clima/rapporti-di-analisi/annuale_pdf/anno_2018.pdf). The median test showed a statistically significant difference in the number of ticks collected per year (*P* = 0.001), as the multivariate regression model demonstrated (*P* = 0.002).

The ticks were taxonomically assigned to ten species of six genera: *Amblyomma*,* Dermacentor*,* Haemaphysalis*,* Hyalomma*,* Ixodes*,* Rhipicephalus* (Table 2). Most ticks were morphologically identified as *Ixodes ricinus* (78.22%). Since some ticks (12.3%) of the genus *Ixodes* were damaged during collection (e.g. missing rostrum), we were unable to classify these specific ticks to the species level. We identified one *Amblyomma parvum* tick from a man who had traveled abroad and was bitten in Brazil, and one *Hyalomma marginatus* tick from a man bitten in Greece*.*

**Table 2:** Tick species collected from humans in the period 2017–2019 and morphologically identified

Tick genus	Tick species	Number of ticks/species
*Ixodes*	*I. ricinus*	1009
	*I. hexagonus*	13
	*I. frontalis*	1
	*I. acuminatus*	1
	*Ixodes* spp*.*	158
Total number *Ixodes* ticks		1182
*Rhipicephalus*	*R. sanguineus*	2
	*Rhipicephalus* spp*.*	6
Total number *Rhipicephalus* ticks		8
*Dermacentor*	*D.marginatus*	6
*Amblyomma*	*A.parvum*	1
*Haemaphysalis*	*H.punctata*	3
*Hyalomma*		1
Not determined		89

Ordered by life stage, the nymph stage was the most frequent life stage (59.8%; 95% confidence interval [CI]: 57.03–62.46), followed by adult females (28.9%; 95% CI: 26.45–31.47), larvae (2.3%; 95% CI: 1.57–3.30) and adult males (0.7%; 95% CI: 0.32–1.32). The median test demonstrated a significantly greater proportion of nymphs (*P* < 0.05), as shown in the multivariate regression model (Table 3).

**Table 3:** Results of the multivariate regression model

Number of ticks (log)	Coefficient	Standard error	*P* value	95% Confidence intterval
Life stage				
Larvae	0.12	0.09	0.19	− 0.06 to 0.30
Nymph	0.25	0.04	0.000*	0.16 to 0.34
Female	0.08	0.05	0.067	− 0.01 to 0.17
Male	− 0.10	0.14	0.467	− 0.36 to 0.17
Year of collection				
2018	0.10	0.03	0.002*	0.04 to 0.17
2019	0.11	0.04	0.002*	0.04 to 0.18
Habitat				
Garden, lawn, park	0.15	0.05	0.002*	0.05 to 0.25
Urban site	− 0.14	0.18	0.442	− 0.49 to 0.21
Country side	− 0.06	0.28	0.834	− 0.61 to 0.49
Woodlands	0.09	0.05	0.057	0.00 to 0.19
Other	− 0.05	0.07	0.524	− 0.19 to 0.10
Bite site				
Trunk	0.07	0.04	0.089	− 0.01 to 0.15
Upper limbs	− 0.07	0.05	0.135	− 0.17 to 0.02
Lower limbs	0.14	0.04	0.001*	0.06 to 0.22
Limbs	0.67	0.23	0.003*	0.22 to 1.12
Other	− 0.02	0.07	0.818	− 0.14 to 0.11
Not determined	− 0.06	0.08	0.465	− 0.22 to 0.10
_cons**	− 0.20	0.07	0.005	− 0.34 to −0.06

*Statistically significant at *P* < 0.05, **Intercept

In terms of the percentage of ticks that tested positive for one or more pathogen according to life stage, we found that 61% of the positive ticks were nymphs, 32% were females, 3% were larvae and 1% were males.

People reported most often receiving a bite on the limbs (43%), followed by the trunk (21%) and the head or neck (especially children) (12%). The median test showed a statistically significant difference for bite site (*P* < 0.05), while the linear regression model demonstrated that the legs were bitten more often than other body sites (*P* = 0.001 for lower limbs, *P* = 0.03 for limbs; Table 3). Most people reported being bitten while walking in the woodlands (40%) or in a garden, lawn, park (46%); the remaining 14% reported being bitten in other locations, such as the beach, train, others. Most tick-bite events occurred while the person was in the woodlands or in garden, lawn or park.

The median test demonstrated the presence of a statistically difference in tick number by habitat (*P* = 0.005). The regression model revealed in a loss of statistical significance for woodlands *P* = 0.057), but not for garden, lawn, park (*P* = 0.002) (Table 3). The least often bitten were the elderly (124 ticks collected in the 3-year period; Table 4), but the difference was not statistically significant (median test, *P* = 0.098).

**Table 4:** Number of ticks by age group (humans) and developmental stage and sex (ticks); *n.d.* not determined

Age group (years)	Life stage					
	Larvae	Nymphs	Female	Male	n.d.	Total
≤ 18	22	321	78	4	25	450
18–50	2	211	99	2	32	346
51–69	4	199	117	2	36	358
≥ 70	2	34	75	0	13	124
Not determined	0	6	4	1	1	12
Total	30	765	369	8	106	1290

A total of 500 ticks (i.e. the total number of ticks collected from the two at-risk populations) underwent biomolecular analysis for pathogen detection. The ticks were analyzed as single sample or pooled; pools were composed of ticks of the same species and collected from the same individual. Overall, 114 ticks (22.8%; 95% CI: 19.19–26.73%) tested positive for one or more pathogens. Most samples testing positive for *Borrelia*, *Rickettsia* or *Anaplasma* (*n* = 71) came from the province of Verbano-Cusio-Ossola (northeast Piedmont), an area with a high tick population, although the prevalence by province was higher for the provinces of Cuneo and Biella (36.84 and 30.77%, respectively) (Table 5).

**Table 5:** Number of ticks which were PCR-positive for one or more pathogens according to pathogen species positivity and location in northwest Italy and of out-of-region

Region	Province	PCR	*Rickettsia* species						*Borrelia* species					
			*R. helvetica*	*R. monacensis*	*R. slovaca*	*R. aeshlimannii*	*Rickettsia spp.*	*A. phagocytophilum*	*B. afzelii*	*B. burgdorferi*	*B. valaisiana*	*B. garinii*	*B. lusitaniae*	*Borrelia spp.*
Piedmont	Biella	13	2	2										
	Cuneo	19	2	2	1		1	1						
	Novara	14	1	1										
	Turin	52	1	4			1	1	6					
	Vercelli	53	4	5			1	1				1		
	Verbano-cusio-ossola	319	19	20	3		2	3	5	1	2	2	4	9
	Alessandria	1												
	Asti	1												
Liguria	Savona	8		1										
	La spezia	1												1
Not determined		6	2											1
Out-of-region		13				1								

Of these 500 ticks analyzed, 76 were positive for *Rickettsia* spp. (15%; 95% CI: 12.17–18.65%), and sequencing analysis identified the species as *R. helvetica* (*n* = 31; *I. ricinus*: *n* = 3 larvae, *n* = 16 nymphs, *n* = 7 adult females; *Ixodes* sp.: *n* = 4 nymphs, *n* = 1 adult female); *R. monacensis* (*n* = 35; *I. ricinus*: *n* = 23 nymphs, *n* = 11 adult females; *Ixodes* spp. *n* = 1 adult female); *R. slovaca* (*n* = 4; *I. ricinus*: *n* = 2 nymph, *n* = 1 adult female; *Dermacentor marginatus* (*n* = 1 adult female)); *R. aeshlimannii* (*n* = 1 *Rhipicephalus sanguineu*s adult male); *Rickettsia* spp. (*n* = 5; *I. ricinus*: *n* = 5 nymphs). Six ticks were positive for *Anaplasma phagocytophilum* (*n* = 6; *I. ricinus* nymphs; 1.2%; 95% CI: 0.44–2.59%).

In comparison, 32 tick samples were positive for *Borrelia burgdorferi* (*s.l.*) (6.4%; 95% CI: 4.44-8.95%). Sequencing and BLAST identified the genospecies as: *B. afzelii* (*n* = 11; *I. ricinus*: *n* = 6 nymphs, *n* = 3 adult females; *Ixodes* spp.: *n* = 2 adult females); *B. burgdorferi *(*s.s.*) (*n* = 1; *I. ricinus* nymph), *B. garinii* (*n* = 3; *I. ricinus* nymph), *B. lusitaniae* (*n* = 4; *I. ricinus*: *n* = 1 nymph, *n* = 3 adult females) and *B. valaisiana* (*n* = 2 adult females) (Tables 6, 7).

**Table 6:** Number of tick species identified and number and percentage of positive samples of each tick species for one or more pathogens

Tick species (*n*)	Number of ticks positive for pathogens (%)		
	*Rickettsia* spp.	*Borrelia* spp.	*Anaplasma* spp.
*Ixodes ricinus* (1009)	68	23	6
*Ixodes hexagonus* (13)	0	0	0
*Ixodes frontalis* (1)	0	0	0
*Ixodes acuminatus*(1)	0	0	0
*Ixodes spp.* (158)	6	2	0
*Rhipicephalus sanguineus* (2)	1	0	0
*Rhiphicephalus* spp. (6)	0	0	0
*Dermacentor marginatus* (6)	1	0	0
*Amblyomma parvum* (1)	0	0	0
*Haemaphisalys punctata* (3)	0	0	0
*Hyalomma marginatum* (1)	0	0	0
Not determined (89)	0	7	0
Total (1290)	Total 76	Total 32	Total 6

**Table 7:** Number and percentage of ticks that tested positive for different pathogens.

Pathogen (no. of ticks analyzed = 500)	Number of tick samples testing positive (%)
*Rickettsia helvetica*	31 (6.2)
*Rickettsia monacensis*	35 (7.0)
*Rickettsia slovaca*	4 (0.6)
*Rickettsia aeshlimannii*	1 (0.2)
*Rickettsia * spp.	5 (1.0)
*Borrelia afzelii*	11 (2.2)
*Borrelia burgdorferi sl*	1 (0.2)
*Borrelia garinii*	3 (0.6)
*Borrelia lusitaniae*	4 (0.8)
*Borrelia valaisiana*	2 (0.4)
*Borrelia* spp.	11 (2.2)
Total* Borrelia* spp.	32 (6.4)
*Anaplasma phagocytophilum*	6 (1.2)

Two pathogens were detected in four samples (co-infections): *Anaplasma phagocytophilum* + *Rickettsia monacensis*; *Borrelia afzelii* + *R. helvetica*; *B. burgdorferi* (*s.s*) +* R. monacensis*; *B. lusitaniae* +* R. monacensis*.

Seventeen ticks were weakly positive by PCR, and sequencing was not successful; only the pathogen genera were assigned in these cases.

## Discussion

The main findings of the present study are the prevalence and the distribution of tick species and tick-borne pathogens collected from humans in northwest Italy. Most samples were collected from children, often bitten by nymphs and females, whereas fewer samples came from the elderly. Males ticks (*I. ricinus*) feed as larvae and nymphs but take only occasional, small blood meals as adults. Their occurrence in humans is uncommon [39]. Almost all pathogens were detected in *I. ricinus*, the primary vector of TBD, as reported elsewhere [3, 17]. Numerous studies have been conducted in northeast Italy, a region that is under continuous surveillance for ticks and associated TBDs [5, 8, 13], whereas surveillance in northwest Italy has been patchy and somewhat limited in terms of defining TBD epidemiology [10, 30].

Here, we report data from a 3-year survey of ticks and tick-borne pathogens circulating throughout northwest Italy. Our data show a wide distribution of tick-borne pathogens and a considerable prevalence in some provinces, such as Cuneo (southwest Piedmont), Biella and Verbano-Cusio-Ossola (both northeast Piedmont). The number of cases of TBD is likely associated with the high number of tick populations; further analysis of climatic and host factors is needed to better understand the dynamics of tick populations and their pathogens.

*Ixodes ricinus*, one of the most abundant tick species in Italy, is an important vector of infection. In our study, it ranked as the most frequently detected and most common species collected from humans. This finding is shared by a previous study reporting that *I. ricinus* was widespread in woodland areas of northwest Italy, where *Ixodes* ticks find optimal conditions of temperature (20–23 °C) and relative humidity (85–98%) for their development [40]. Despite their small size, the majority of ticks were nymphs (60%), in line with findings reported by Otranto et al. [28], and thus may be easily overlooked [41].

*Rickettsia* was the most frequently detected pathogen genus, with 76 *Rickettsia*-positive ticks out of the 500 tested (prevalence 15%; 95% CI: 12.17–18.65%); this finding is in line with a previous study carried out in Italy [6]. *Rickettsia conorii conorii*, comprising a variety of genospecies, is considered to be the main etiologic agent of MSF. MSF is widely distributed through southern Europe, Africa and the Middle East, where it is an emerging or re-emerging disease in some areas. Sequencing of genetic markers has led to the molecular characterization of strains and the identification of many new *Rickettsia* spp. or subspecies within the spotted fever group involved in human rickettsiosis [19]. In our study, sequencing revealed four *Rickettsia* species (*R. helvetica*,* R. monacensis*,* R. slovaca*, *R. aeschlimanni*). *Rickettsia monacensis* was first isolated in Germany and is now widespread throughout Europe in *I. ricinus* tick vectors. In Italy, it was isolated in Sardinia [20], Sicily and Liguria [28] from *I. ricinus* and *Dermacentor marginatus*.

Previous studies have associated *R. helvetica* with perimyocarditis [24], fever and skin rash [25] and, more recently, with subacute meningitis [26]. Its main carrier is *I. ricinus* and its prevalence varies across Europe [27]. To date, it has been isolated in north Italy (Liguria, Veneto, Friuli-Venezia-Giulia, Trentino) from *I. ricinus* [28].

On the other hand,* R. slovaca* was previously largely isolated in the south of Italy (Sicily, Basilicata, Puglia) and in Liguria from *R. sanguineus* and *D. marginatus* [4, 28]. It is implicated in tick-borne lymphadenopathy (TIBOLA) and* Dermacentor*-borne necrosis erythema and lymphadenopathy (DEBONEL). Here we report one case of *R. slovaca* infection in *D. marginatus*; it was also identified in two *I. ricinus* ticks.

*Rickettsia aeshlimanni* has been identified in tick species *Hyalomma marginatum* and *I. ricinus* [29] and potentially in new species (*D. marginatus* and *H. lusitanicum*). In Europe, *R. aeshlimanni* mainly infects ticks of the genus *Hyalomma*; the pathogenicity of this bacterium is not well understood, although MSF-like lesions have been reported [15]. It has been isolated in Sicily, Latium and Tuscany from *H. marginatum* [4, 33]. Here, we report for the first time the isolation of *R. aeshlimannii* from *Rhipicephalus sanguineus* in Capoliveri (Tuscany 2018)*.* We also found* Anaplasma phagocytophilum* in *I. ricinus*.

Regarding *Borrelia burgdorferi* (*s.l.*), our findings indicate a low prevalence. Sequencing identified five genomic groups of the *B. burgorferi* (*s.l.*) complex: *B. afzelii*,* B. burgdorferi* (*s.s.*),* B. garinii*,* B. lusitaniae* and *B. valaisiana.* Three of these genomic groups are considered pathogenic for humans: *B. afzelii* is mainly associated with skin manifestations; *B. burgdorferi * (*s.s.*) is the agent of Lyme arthritis; and *B. garinii* is the only species linked to neuroborreliosis [31]. *Borrelia lusitaniae* and *B. valaisiana* are of uncertain pathogenicity. Our study adds new knowledge about the distribution of the genospecies of the *B. burgdorferi* complex, which is a pathogen of ecological and epidemiological interest.

**Fig. 1:**
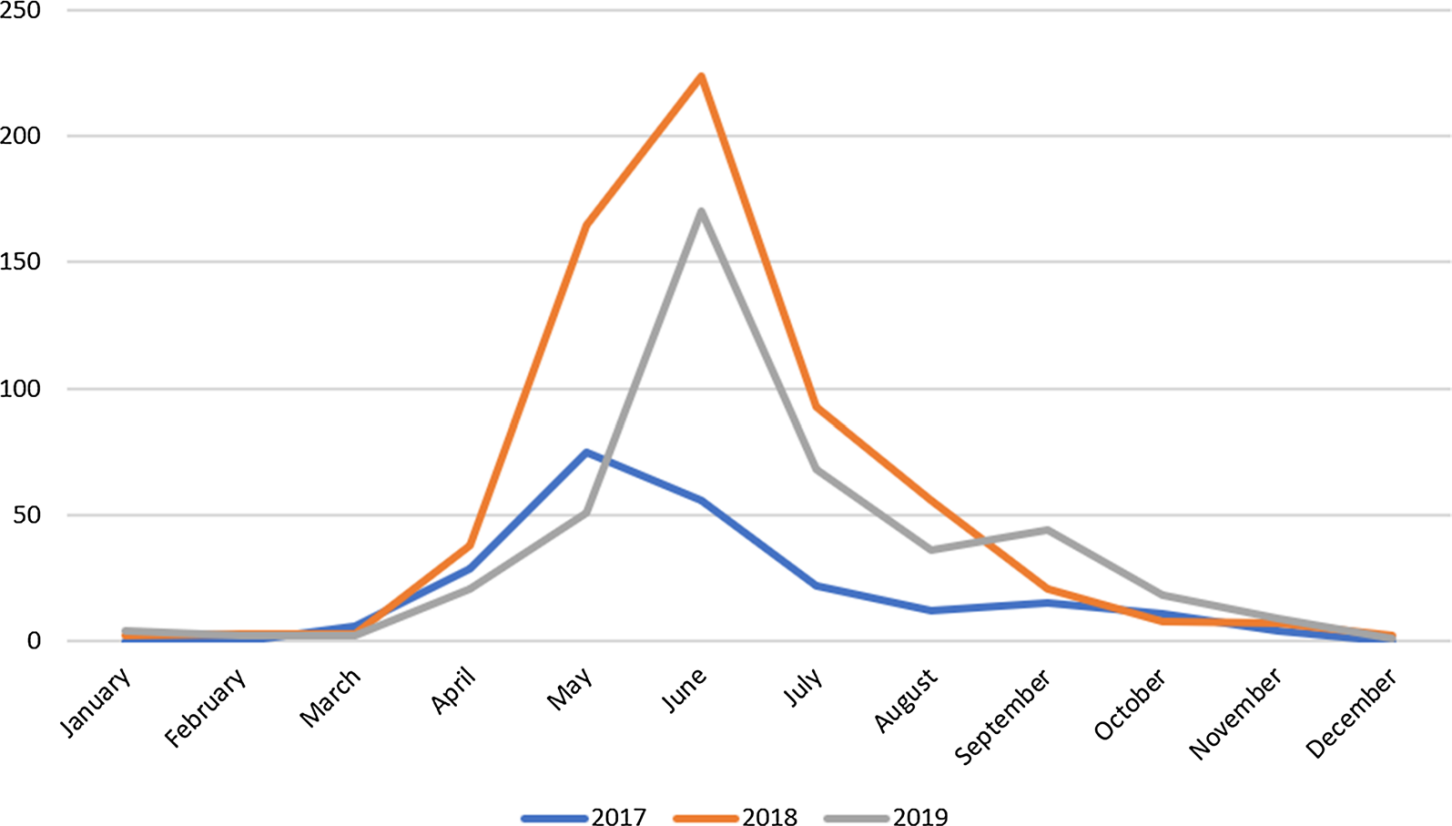
Number of ticks collected by month during 2017–2019

The ecological and climatic conditions of northwest Italy are conducive to tick persistence and spread, which means greater exposure of the population to tick bites. Peak tick activity occurs in the spring and summer when the climatic conditions are favorable for tick reproduction, and reports of tick-bite events increase in these seasons as more people visit woodlands for recreation (Fig. 1). Our data show variation in the number of ticks collected per year (range: 239–624 samples), which probably reflects climatic conditions, since sampling was irregular during the course of the 3 years. While 2017 was quite a dry year, rainfall in the spring and summer of 2018 was abundant (May 2018 was recorded as the seventh wettest month since 1958 (http://www.arpa.piemonte.it/rischinaturali/bacheca-archivio/bacheca-archivio.html). The summer temperatures, which remained within the average range (http://www.arpa.piemonte.it/rischinaturali/widget/servizi-siti-web-regione.html?delta=0) created ideal conditions for greater parasitic environmental pressure. The result was an increase in tick-bite events in humans between May and July 2018. Similarly, the highest number of tick-bite events was recorded in May and June of 2019. The early peak in collected ticks recorded for May of 2017 probably stemmed from the warm and wet weather conditions in the area from February to April (https://www.arpa.piemonte.it/rischinaturali/tematismi/clima/rapporti-di-analisi/annuale_pdf/anno_2017.pdf). Most tick-bite events occurred during a walk in the woodlands, which is an ideal habitat for *I. ricinus*. Numerous events were recorded also in urban areas according to the data collected by physicians.

The identification of tick pathogens in 24% of the samples underlines the importance of surveillance, prevention and correct diagnosis in humans. The increase in the prevalence and transmission of TBD raises concern for public health. The geographic spread of tick species driven by changes in micro- and macroclimate, human activities, land use, vector population growth and many other factors has brought about an increase in TBD. As we continue to discover new species of bacteria, it is essential to monitor the emergence of new and existing pathogens. Tick surveillance and tracking can enhance our understanding of tick spread and ecology and identify areas of risk for disease transmission.

**Fig. 2:**
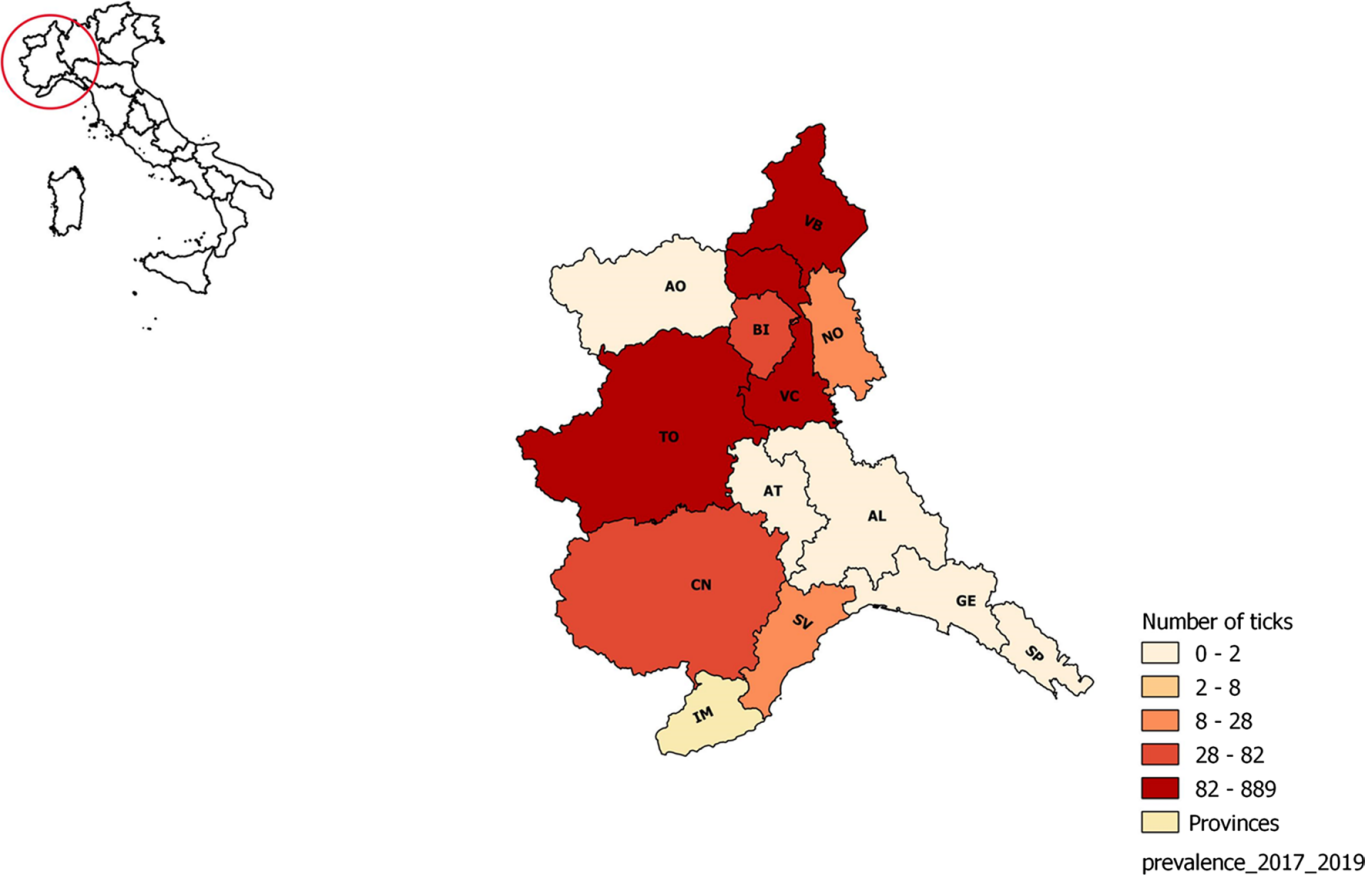
Map showing the geographic distribution (%) of ticks collected in northwest Italy

It is also important to raise awareness in the population as personal protective strategies can help in the prevention of TBD. Exposure to ticks may also result from exposure to domestic and companion animals that bring ticks into the house. Tick prevention using repellents and tick checks after domestic animal exposure are simple risk reduction measures. As novel tick-transmitted pathogens are discovered and emerge in geographic regions, our ability to detect, describe and understand this growing public health threat must step up to meet the challenge (Fig. 2).

## Conclusions

The risk of TBD in humans is associated with local tick abundance, infection prevalence, density of vertebrate reservoir hosts, and climate change. Our data show that humans bitten by ticks in northwest Italy are at risk of infection from diverse pathogens. Co-infected ixodids were also detected, indicating that more than one pathogen may be transmitted by the same tick bite with potential for multiple infections in humans. Local information campaigns are essential for prevention and protection against TBD. Further analysis of these factors may help in assessing risks and guide the implementation of public health policies against TBD.

## Data Availability

The datasets used and/or analyzed during the current study are available from the corresponding author on reasonable request.

## Abbreviations

IZS PLVA: Istituto Zooprofilattico Sperimentale of Piedmont, Liguria and Valle d’Aosta
MSF: Mediterranean Spotted Fever
TBDs: Tick-borne diseases
TBE: Tick-borne encephalitis
